# A second monoclinic polymorph of 2-(diformyl­methyl­idene)-3,3-dimethyl-2,3-dihydro-1*H*-indole

**DOI:** 10.1107/S1600536809038495

**Published:** 2009-09-30

**Authors:** Hamid Khaledi, Siti Munirah Saharin, Hapipah Mohd Ali, Ward T. Robinson, Mahmood A. Abdulla

**Affiliations:** a Department of Chemistry, University of Malaya, 50603 Kuala Lumpur, Malaysia; b Department of Molecular Medicine, University of Malaya, 50603 Kuala Lumpur, Malaysia

## Abstract

The crystal structure of the title compound, C_13_H_13_NO_2_, is a polymorph of the structure first reported by Helliwell *et al.* [*Acta Cryst.* (2006), E**62**, o737-o738]. It is also monoclinic (space group *P*2_1_/*c*), but with completely different cell constants. The mol­ecular conformations of these polymorphs differ by a 180° rotation of one formyl group. The present mol­ecule is planar [maximum deviation 0.089 (2) Å] with the exception of the two methyl groups which lie on either side of the plane. There are strong intra- and inter­molecular N—H⋯O hydrogen bonds. The latter link pairs of mol­ecules across crystallographic centers of symmetry. Two aldehyde O atoms are brought close together [2.896 (4) Å in this arrangement but are not hydrogen bonded. In the earlier polymorph, one formyl group is rotated by 180° to yield inter­molecular hydrogen bonding and an infinite polymeric chain. The other formyl group is involved in the same intra­molecular hydrogen bonding as has been found here.

## Related literature

For the crystal structure of the other polymorph, see: Helliwell *et al.* (2006 ▶). For a discussion of crystal growth conditions that can affect the occurrence of polymorphs, see: Hulliger *et al.* (1994 ▶). For chemistry involving 2-(diformyl­methyl­idene)-3,3-dimethyl-2,3-dihydro-1*H*-indole, see: Baradarani *et al.* (2006 ▶).
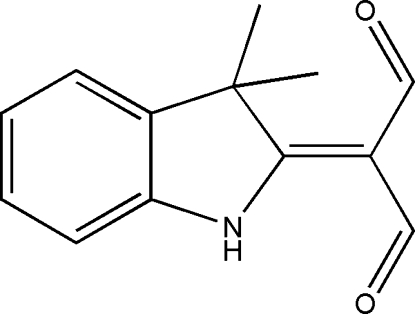


## Experimental

### 

#### Crystal data

C_13_H_13_NO_2_
*M* *_r_* = 215.24
Monoclinic, 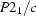

*a* = 6.9877 (10) Å
*b* = 18.688 (3) Å
*c* = 8.2154 (12) Å
β = 90.291 (3)°
*V* = 1072.8 (3) Å^3^
*Z* = 4
Mo *K*α radiation
μ = 0.09 mm^−1^
*T* = 103 K
0.57 × 0.37 × 0.03 mm

#### Data collection

Bruker APEXII CCD diffractometer
Absorption correction: multi-scan (*SADABS*; Sheldrick, 1996 ▶) *T*_min_ = 0.950, *T*_max_ = 0.997
4763 measured reflections
1865 independent reflections
1123 reflections with *I* > 2σ(*I*)
*R*_int_ = 0.072

#### Refinement

*R*[*F* ^2^ > 2σ(*F* ^2^)] = 0.044
*wR*(*F* ^2^) = 0.103
*S* = 0.92
1865 reflections
151 parameters
H atoms treated by a mixture of independent and constrained refinement
Δρ_max_ = 0.26 e Å^−3^
Δρ_min_ = −0.22 e Å^−3^

### 

Data collection: *APEX2* (Bruker, 2007 ▶); cell refinement: *SAINT* (Bruker, 2007 ▶); data reduction: *SAINT*; program(s) used to solve structure: *SHELXS97* (Sheldrick, 2008 ▶); program(s) used to refine structure: *SHELXL97* (Sheldrick, 2008 ▶); molecular graphics: *SHELXTL* (Sheldrick, 2008 ▶); software used to prepare material for publication: *SHELXL97* and *publCIF* (Westrip, 2009 ▶).

## Supplementary Material

Crystal structure: contains datablocks I, global. DOI: 10.1107/S1600536809038495/om2278sup1.cif

Structure factors: contains datablocks I. DOI: 10.1107/S1600536809038495/om2278Isup2.hkl

Additional supplementary materials:  crystallographic information; 3D view; checkCIF report

## Figures and Tables

**Table 1:** Hydrogen-bond geometry (Å, °)

*D*—H⋯*A*	*D*—H	H⋯*A*	*D*⋯*A*	*D*—H⋯*A*
N1—H1⋯O1	0.93 (3)	1.93 (3)	2.642 (3)	132 (2)
N1—H1⋯O1^i^	0.93 (3)	2.19 (3)	2.946 (2)	138 (2)

Symmetry code: (i) 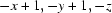.

**Fig. 1.**
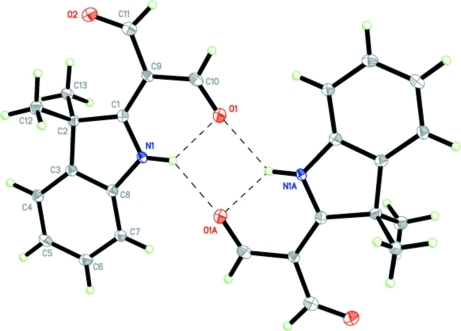
A perspective drawing of two molecules of the title compound showing dimerization through intermolecular H-bonds. Displacement ellipsoids are drawn at the 30% probability level. Symmetry code: A = 1-x, 1-y, -z

## supplementary crystallographic information

### Experimental

A solution of trimethylindolenine, (5.57 g, 35 mmol), in anhydrous dimethylformamide (15 ml) was cooled in an ice bath. A solution of phosphoryl chloride (10 ml) in dimethylformamide (15 ml) was added dropwise with stirring over a period of 1 h at below 283 K. The cooling bath was removed and the reaction mixture was stirred at 363 K for 2 h. The resulting solution was poured onto ice water (400 ml), the pH was adjusted to 9.0 by the addition of aqueous NaOH (35%) whereupon the solid product was precipitated. It was filtered, washed with hot water, dried and recrystallized from n-hexane/ethyl acetate to give a yellow solid in 4.35 g, 58% yield. Further recrystallization, from ethanol/water (3:1 *v/v*), led to a mixture of lath and prismatic habits. The prism cell dimensions confirmed the form reported earlier but the laths appeared new.

### Refinement

C-bound hydrogen atoms were placed at calculated positions (C–H = 0.95–0.98 Å) and refined as riding with U(H) = 1.2–1.5 times *U*_eq_(C). The N-bound hydrogen atom was located from a difference map, and freely refined to give a bond length of 0.93 (3) Å.

### Crystal data

**Table:**

C_13_H_13_NO_2_	*F*(000) = 456
*M**_r_* = 215.24	*D*_x_ = 1.333 Mg m^−^^3^
Monoclinic, *P*2_1_/*c*	Mo *K*α radiation, λ = 0.71073 Å
Hall symbol: -P 2ybc	Cell parameters from 864 reflections
*a* = 6.9877 (10) Å	θ = 2.2–24.7°
*b* = 18.688 (3) Å	µ = 0.09 mm^−^^1^
*c* = 8.2154 (12) Å	*T* = 103 K
β = 90.291 (3)°	Lath, yellow
*V* = 1072.8 (3) Å^3^	0.57 × 0.37 × 0.03 mm
*Z* = 4	

### Data collection

**Table:**

Bruker APEXII CCD diffractometer	1865 independent reflections
Radiation source: fine-focus sealed tube	1123 reflections with *I* > 2σ(*I*)
graphite	*R*_int_ = 0.072
ω scans	θ_max_ = 25.1°, θ_min_ = 2.2°
Absorption correction: multi-scan (*SADABS*; Sheldrick, 1996)	*h* = −5→8
*T*_min_ = 0.950, *T*_max_ = 0.997	*k* = −21→22
4763 measured reflections	*l* = −9→9

### Refinement

**Table:**

Refinement on *F*^2^	Primary atom site location: structure-invariant direct methods
Least-squares matrix: full	Secondary atom site location: difference Fourier map
*R*[*F*^2^ > 2σ(*F*^2^)] = 0.044	Hydrogen site location: inferred from neighbouring sites
*wR*(*F*^2^) = 0.103	H atoms treated by a mixture of independent and constrained refinement
*S* = 0.92	*w* = 1/[σ^2^(*F*_o_^2^) + (0.0424*P*)^2^] where *P* = (*F*_o_^2^ + 2*F*_c_^2^)/3
1865 reflections	(Δ/σ)_max_ < 0.001
151 parameters	Δρ_max_ = 0.26 e Å^−^^3^
0 restraints	Δρ_min_ = −0.22 e Å^−^^3^

### Special details

**Table:**

Geometry. All esds (except the esd in the dihedral angle between two l.s. planes) are estimated using the full covariance matrix. The cell esds are taken into account individually in the estimation of esds in distances, angles and torsion angles; correlations between esds in cell parameters are only used when they are defined by crystal symmetry. An approximate (isotropic) treatment of cell esds is used for estimating esds involving l.s. planes.
Refinement. Refinement of *F*^2^ against ALL reflections. The weighted *R*-factor *wR* and goodness of fit *S* are based on *F*^2^, conventional *R*-factors *R* are based on *F*, with *F* set to zero for negative *F*^2^. The threshold expression of *F*^2^ > σ(*F*^2^) is used only for calculating *R*-factors(gt) *etc*. and is not relevant to the choice of reflections for refinement. *R*-factors based on *F*^2^ are statistically about twice as large as those based on *F*, and *R*- factors based on ALL data will be even larger.

### Fractional atomic coordinates and isotropic or equivalent isotropic displacement parameters (Å^2^)

**Table:**

	*x*	*y*	*z*	*U*_iso_*/*U*_eq_	
O1	0.4035 (3)	0.49657 (9)	0.1554 (2)	0.0308 (5)	
O2	0.4628 (3)	0.65460 (9)	0.6032 (2)	0.0346 (5)	
N1	0.7133 (3)	0.57750 (10)	0.1457 (2)	0.0192 (5)	
H1	0.637 (4)	0.5445 (14)	0.092 (3)	0.054 (9)*	
C1	0.6569 (3)	0.60337 (11)	0.2893 (3)	0.0195 (5)	
C2	0.8061 (3)	0.65754 (12)	0.3488 (3)	0.0199 (6)	
C3	0.9518 (3)	0.65385 (12)	0.2146 (3)	0.0197 (6)	
C4	1.1227 (3)	0.68961 (12)	0.1934 (3)	0.0225 (6)	
H4	1.1673	0.7228	0.2725	0.027*	
C5	1.2281 (4)	0.67584 (12)	0.0535 (3)	0.0253 (6)	
H5	1.3459	0.7002	0.0373	0.030*	
C6	1.1650 (4)	0.62761 (12)	−0.0621 (3)	0.0259 (6)	
H6	1.2405	0.6190	−0.1560	0.031*	
C7	0.9931 (3)	0.59154 (12)	−0.0434 (3)	0.0221 (6)	
H7	0.9479	0.5585	−0.1226	0.027*	
C8	0.8906 (3)	0.60609 (11)	0.0965 (3)	0.0196 (5)	
C9	0.4879 (3)	0.57998 (12)	0.3639 (3)	0.0192 (6)	
C10	0.3723 (4)	0.52662 (12)	0.2862 (3)	0.0258 (6)	
H10	0.2591	0.5128	0.3415	0.031*	
C11	0.4105 (4)	0.60552 (13)	0.5160 (3)	0.0257 (6)	
H11	0.3018	0.5801	0.5539	0.031*	
C12	0.7221 (4)	0.73372 (12)	0.3575 (3)	0.0262 (6)	
H12A	0.8248	0.7679	0.3812	0.039*	
H12B	0.6261	0.7359	0.4438	0.039*	
H12C	0.6621	0.7458	0.2530	0.039*	
C13	0.8926 (3)	0.63454 (12)	0.5131 (3)	0.0241 (6)	
H13A	0.9436	0.5859	0.5039	0.036*	
H13B	0.7932	0.6357	0.5968	0.036*	
H13C	0.9961	0.6674	0.5433	0.036*	

### Atomic displacement parameters (Å^2^)

**Table:**

	*U*^11^	*U*^22^	*U*^33^	*U*^12^	*U*^13^	*U*^23^
O1	0.0343 (11)	0.0320 (10)	0.0261 (10)	−0.0086 (9)	−0.0002 (8)	−0.0040 (8)
O2	0.0367 (12)	0.0377 (11)	0.0295 (10)	−0.0023 (9)	0.0058 (9)	−0.0071 (9)
N1	0.0143 (12)	0.0217 (11)	0.0215 (11)	−0.0051 (9)	0.0010 (9)	−0.0019 (9)
C1	0.0213 (15)	0.0178 (12)	0.0192 (12)	0.0006 (11)	−0.0039 (11)	0.0024 (10)
C2	0.0234 (15)	0.0187 (12)	0.0176 (12)	−0.0011 (11)	0.0010 (11)	−0.0001 (10)
C3	0.0223 (15)	0.0183 (12)	0.0185 (12)	0.0021 (11)	0.0003 (11)	0.0028 (10)
C4	0.0205 (15)	0.0230 (13)	0.0239 (13)	−0.0023 (11)	−0.0024 (11)	0.0018 (10)
C5	0.0198 (15)	0.0262 (14)	0.0299 (14)	−0.0006 (11)	0.0033 (12)	0.0067 (11)
C6	0.0292 (17)	0.0253 (13)	0.0233 (13)	0.0041 (12)	0.0055 (12)	0.0039 (11)
C7	0.0227 (15)	0.0237 (13)	0.0201 (12)	−0.0017 (11)	0.0009 (11)	0.0000 (10)
C8	0.0208 (14)	0.0157 (12)	0.0223 (12)	0.0028 (11)	0.0000 (11)	0.0031 (10)
C9	0.0154 (14)	0.0216 (13)	0.0204 (12)	−0.0019 (11)	−0.0005 (11)	0.0033 (10)
C10	0.0235 (16)	0.0267 (14)	0.0271 (14)	−0.0004 (12)	0.0010 (12)	0.0077 (11)
C11	0.0207 (15)	0.0278 (14)	0.0286 (14)	0.0045 (12)	−0.0003 (12)	0.0040 (12)
C12	0.0313 (16)	0.0215 (13)	0.0259 (13)	0.0023 (12)	0.0020 (12)	−0.0006 (10)
C13	0.0214 (15)	0.0301 (14)	0.0207 (13)	−0.0033 (11)	−0.0011 (11)	0.0016 (10)

### Geometric parameters (Å, °)

**Table:**

O1—C10	1.233 (3)	C5—H5	0.9500
O2—C11	1.219 (3)	C6—C7	1.387 (3)
N1—C1	1.336 (3)	C6—H6	0.9500
N1—C8	1.410 (3)	C7—C8	1.384 (3)
N1—H1	0.93 (3)	C7—H7	0.9500
C1—C9	1.403 (3)	C9—C10	1.431 (3)
C1—C2	1.531 (3)	C9—C11	1.445 (3)
C2—C3	1.506 (3)	C10—H10	0.9500
C2—C13	1.538 (3)	C11—H11	0.9500
C2—C12	1.542 (3)	C12—H12A	0.9800
C3—C4	1.380 (3)	C12—H12B	0.9800
C3—C8	1.384 (3)	C12—H12C	0.9800
C4—C5	1.392 (3)	C13—H13A	0.9800
C4—H4	0.9500	C13—H13B	0.9800
C5—C6	1.380 (3)	C13—H13C	0.9800
			
C1—N1—C8	112.3 (2)	C6—C7—H7	121.7
C1—N1—H1	119.3 (17)	C3—C8—C7	123.3 (2)
C8—N1—H1	128.4 (17)	C3—C8—N1	108.2 (2)
N1—C1—C9	121.7 (2)	C7—C8—N1	128.5 (2)
N1—C1—C2	108.5 (2)	C1—C9—C10	119.8 (2)
C9—C1—C2	129.8 (2)	C1—C9—C11	126.5 (2)
C3—C2—C1	101.40 (18)	C10—C9—C11	113.8 (2)
C3—C2—C13	111.46 (19)	O1—C10—C9	127.1 (2)
C1—C2—C13	111.06 (18)	O1—C10—H10	116.4
C3—C2—C12	109.57 (18)	C9—C10—H10	116.4
C1—C2—C12	111.51 (19)	O2—C11—C9	130.1 (2)
C13—C2—C12	111.43 (18)	O2—C11—H11	114.9
C4—C3—C8	119.2 (2)	C9—C11—H11	114.9
C4—C3—C2	131.2 (2)	C2—C12—H12A	109.5
C8—C3—C2	109.6 (2)	C2—C12—H12B	109.5
C3—C4—C5	118.4 (2)	H12A—C12—H12B	109.5
C3—C4—H4	120.8	C2—C12—H12C	109.5
C5—C4—H4	120.8	H12A—C12—H12C	109.5
C6—C5—C4	121.4 (2)	H12B—C12—H12C	109.5
C6—C5—H5	119.3	C2—C13—H13A	109.5
C4—C5—H5	119.3	C2—C13—H13B	109.5
C5—C6—C7	121.0 (2)	H13A—C13—H13B	109.5
C5—C6—H6	119.5	C2—C13—H13C	109.5
C7—C6—H6	119.5	H13A—C13—H13C	109.5
C8—C7—C6	116.7 (2)	H13B—C13—H13C	109.5
C8—C7—H7	121.7		

### Hydrogen-bond geometry (Å, °)

**Table:**

*D*—H···*A*	*D*—H	H···*A*	*D*···*A*	*D*—H···*A*
N1—H1···O1	0.93 (3)	1.93 (3)	2.642 (3)	132 (2)
N1—H1···O1^i^	0.93 (3)	2.19 (3)	2.946 (2)	138 (2)

Symmetry codes: (i) −*x*+1, −*y*+1, −*z*.
